# Spatial Distribution of Selected Chemical Cell Wall Components in the Embryogenic Callus of *Brachypodium distachyon*

**DOI:** 10.1371/journal.pone.0167426

**Published:** 2016-11-28

**Authors:** Alexander Betekhtin, Magdalena Rojek, Anna Milewska-Hendel, Robert Gawecki, Jagna Karcz, Ewa Kurczynska, Robert Hasterok

**Affiliations:** 1 Department of Plant Anatomy and Cytology, Faculty of Biology and Environmental Protection, University of Silesia in Katowice, Katowice, Poland; 2 Department of Cell Biology, Faculty of Biology and Environmental Protection, University of Silesia in Katowice, Katowice, Poland; 3 Scanning Electron Microscopy Laboratory, Faculty of Biology and Environmental Protection, University of Silesia in Katowice, Katowice, Poland; United States Department of Agriculture, UNITED STATES

## Abstract

*Brachypodium distachyon* L. Beauv. (Brachypodium) is a species that has become an excellent model system for gaining a better understanding of various areas of grass biology and improving plant breeding. Although there are some studies of an *in vitro* Brachypodium culture including somatic embryogenesis, detailed knowledge of the composition of the main cell wall components in the embryogenic callus in this species is missing. Therefore, using the immunocytochemical approach, we targeted 17 different antigens of which five were against the arabinogalactan proteins (AGP), three were against extensins, six recognised pectic epitopes and two recognised hemicelluloses. These studies were complemented by histological and scanning electron microscopy (SEM) analyses. We revealed that the characteristic cell wall components of Brachypodium embryogenic calli are AGP epitopes that are recognised by the JIM16 and LM2 antibodies, an extensin epitope that is recognised by the JIM11 antibody and a pectic epitopes that is recognised by the LM6 antibody. Furthermore, we demonstrated that AGPs and pectins are the components of the extracellular matrix network in Brachypodium embryogenic culture. Additionally, SEM analysis demonstrated the presence of an extracellular matrix on the surface of the calli cells. In conclusion, the chemical compositions of the cell walls and ECMSN of Brachypodium callus show spatial differences that correlate with the embryogenic character of the cells. Thus, the distribution of pectins, AGPs and hemicelluloses can be used as molecular markers of embryogenic cells. The presented data extends the knowledge about the chemical composition of the embryogenic callus cells of Brachypodium.

## Introduction

*Brachypodium distachyon* L. Beauv. (Brachypodium), a member of the Pooideae subfamily, is a wild annual grass species that has a wide range of occurrence. Although its natural habitats are found in regions of the Mediterranean basin, the Middle East, south-west Asia and north-east Africa, due to its introduction beyond its natural range, populations of this species have also been observed in North and South America, Australia and Western Europe [1]. Brachypodium is closely related to many temperate zone key cereals, such as wheat, barley, rye and oats as well as forage grasses. It has many useful biological traits, for example a small nuclear genome, small stature, rapid life cycle, the ability to self-pollinate and simple growth requirements, which along with the diverse germplasm resources and well-developed research infrastructure make this species an excellent model system for both a better understanding of grass biology and improving plant breeding, including the faster domestication of emerging crops [2, 3]. Recently, the main fields of research on Brachypodium have been extensively reviewed in [4].

Brachypodium is receptive to *in vitro* manipulation and transformation [5, 6] and its T-DNA mutagenesis is based on the transformation of its embryogenic callus lines [7]. Although it was demonstrated that a high-efficiency transformation callus can also be obtained from whole seeds, immature embryos are the most suitable explant for callus induction in Brachypodium [8, 9]. These embryos are highly susceptible to the stimulatory conditions of an *in vitro* culture, which results in the first callus clusters being observed after only a week [10]. Such a callus is of a high quality and regeneration potential, which makes it a preferred target for genetic transformation [7]. The embryogenic callus of Brachypodium is typically induced using a Murashige & Skoog (SM) or Linsmaier & Skoog (LS) medium that is supplemented with different concentrations of 2,4-dichlorophenoxyacetic acid (2,4-D). The regeneration of fully developed, fertile green plants is quite easy to achieve on common media, e.g. MS supplemented with kinetin or 6-benzyloaminpurine (BAP), which means that Brachypodium has no unusual requirements for regeneration [5, 7].

Somatic embryogenesis (SE) is a remarkable phenomenon that enables plant somatic cells to develop into the structures that in terms of both their morphology and physiology resemble zygotic embryos [11]. It is divided into three main stages: (i) the induction of the embryogenic cells/callus, (ii) the development of the somatic embryos and (iii) the conversion of the somatic embryos into fully regenerated plants [12, 13]. SE has been well characterised in many dicot species, especially in *Arabidopsis thaliana* [14, 15] as well as in several monocots, including grasses [16, 17]. Although the protocols for embryogenic callus induction in Brachypodium were developed some time ago, there is no information about the morphology, histology and biochemistry of SE in this species.

A dynamic reorganisation of the cell wall components is essential during SE [18]. Embryogenic callus cells differ significantly from non-embryogenic cells in several prominent structural and biochemical aspects, such as the cell size, characteristic ultrastructure and compartmentation of the organelles, the capacity to synthesise specific proteins and cell wall components [19, 20]. These modifications are usually associated with the degradation, deposition and synthesis of new macromolecules, such as hemicelluloses, arabinogalactan proteins (AGPs) and pectins [21]. AGPs are plant-specific macromolecules that belong to the subfamily of hydroxyproline-rich glycoproteins (HRGPs). They can function as signalling molecules and regulate cell differentiation as well as vegetative and reproductive organ development [22, 23]. In turn, pectins are acidic polysaccharides and are a major class of the structural molecules of primary the cell walls in land plants and are responsible for the determination of their mechanical properties [24]. Both AGPs and pectins have been shown to play key roles in the regulation of SE initiation and the further development of somatic embryos. It has been suggested that they may be associated with cell adhesion, signalling and recognition [19, 25]. In addition, these two categories of cell wall compounds are detectable in the fibrillar network outside the outer periclinal walls, which is known as the extracellular matrix surface network (ECMSN) that is characteristic for SE [19, 26]. The detailed significance of this network is still under study, but it has been suggested that it participates in cell adhesion, cell signalling and in the regulation and coordination of somatic embryo development [27]. Hemicelluloses are polysaccharides that are composed of various monosaccharides such as glucose, mannose galactose, arabinose and xylose, which along with cellulose comprise the main component of plant cell wall. The primary function of hemicelluloses is maintaining the cell wall structure and regulating cell growth [28].

This study was undertaken to answer the intriguing question about the chemical composition of embryogenic callus cells and to determine whether the ECMSN is present in the Brachypodium callus and whether it is linked with the SE process. We used scanning electron microscopy (SEM), light microscopy and histological and immunolabelling techniques to analyse the distribution of selected pectin, AGPs, extensins and hemicelluloses in the cell walls, the internal cell compartments and on the callus surface.

## Material and Methods

### Plant material growth and *in vitro* culture conditions

Immature zygotic embryos of *B*. *distachyon* (Brachypodium) line Bd21 were used as the explants for embryogenic callus induction. The immature embryos were isolated from young seeds that were collected from plants growing in pots with soil mixed with vermiculite (3:1, w/v) in a greenhouse at 20±1°C, under a 16/8 h light/dark photoperiod, which was provided by lamps emitting white light at the intensity of 10 000 lx. To ensure synchronised flowering, approximately one-month-old plants were subjected to vernalisation for four weeks at 4°C.

The young seeds were placed on sterile Petri dishes with some sterile distilled water for easier embryo isolation. Embryos were excised under a binocular in a laminar flow hood using sterile preparation needles. Approximately 10–15 immature (0.5–0.7 mm long) embryos were placed on a callus induction medium (CIM, pH 5.8) that consisted of MS salts, vitamins, 30 g/l sucrose, 2.5 mg/l 2,4-D and 8 g/l Select Agar. The Petri dishes with immature embryos were incubated at 28°C in the dark for embryogenic callus induction. The calli with embryogenic complexes were transferred to fresh CIM every three weeks and were also incubated at 28°C in the dark. Analyses were performed on the calli that had been induced on immature embryos after the fourth passage on fresh CIM and the material was fixed on the 7^th^ and 21^st^ day after the transfer. All images were taken using a dissecting microscope SMZ 1500 (Nikon) equipped with a digital camera DS-U2 (Nikon).

### Histological procedures

The calli were fixed in a mixture of 4% (w/v) paraformaldehyde (PFA), 1% (v/v) glutaraldehyde (GA) in phosphate buffered saline (PBS, pH 7.0). To remove the air from the material and to facilitate fixative infiltration, samples were put under a vacuum for 5×15 min. Samples were fixed overnight at 4°C, rinsed with PBS (3×15 min), dehydrated in an ethanol series (10, 30, 50, 70, 90 and 100%; 2×30 min in each) and gradually embedded in LR White resin. The material was cut into 1.5 μm thick sections using an EM UC6 ultramicrotome (Leica Microsystems). Sections were collected on microscopic slides covered with poly-L-lysine. For general histology, sections were stained with a 0.05% (w/v) toluidine blue O for 5 min.

### Immunocytochemistry

Sections were treated for 30 min at room temperature (RT) with a blocking reagent consisting of 2% (v/v) foetal calf serum and 2% (w/v) bovine serum albumin in PBS (pH 7.2). Next, sections were incubated at RT for at least 1.5 h with specific primary monoclonal antibodies (Table 1), diluted 1:20 in a blocking reagent, rinsed 3×10 min with the blocking reagent and incubated at RT for at least 1.5 h with AlexaFluor 488 goat anti-rat IgG (Jackson Immuno-Research Laboratories) diluted 1:100 in the blocking reagent and used as the secondary antibody. After several washes with the blocking reagent and PBS, the sections were stained with 0.01% (w/v) fluorescent brightener 28 (FB28) (Sigma-Aldrich) in PBS for 5 min, then with 2 μg/ml DAPI in PBS (5 min at RT) after which they were thoroughly rinsed with PBS and sterile distilled water. Dried slides were mounted in a Fluoromount (Sigma-Aldrich) anti-fading medium. Negative controls were performed for each antibody used by omitting the primary antibodies. All images were taken using a Zeiss Axio Imager Z2 microscope equipped with an AxioCam Mrm monochromatic camera (Zeiss) with the corresponding software and narrow band filters for AlexaFluor 488 and DAPI.

**Table 1:** The antibodies used in this study, the epitopes they recognise and relevant references.

Antibody	Epitope	References
	*Arabinogalactan proteins (AGPs)*	
MAC207	Carbohydrate part of arabinogalactan protein	[67]
JIM4	Carbohydrate part of arabinogalactan protein	[68]
JIM8	Arabinogalactan protein	[69]
JIM13	Arabinogalactan/arabinogalactan protein	[43]
JIM16	Arabinogalactan/arabinogalactan protein	[43]
LM2	Carbohydrate part of arabinogalactan protein	[70]
	*Extensins*	
LM1	Extensin	[71]
JIM11	Extensin	[72]
JIM12	Extensin	[72]
	*Pectins*	
LM19	Homogalacturonan (HG) domain in pectic polysaccharides, which recognises a range of HGs with preferential binding to unesterified HGs	[73]
LM20	HG domain in pectic polysaccharides, which requires methyl esters for recognition of HG and does not bind to unesterified HG	[73]
LM13	(1–5)-a-L-arabinan (linear)	[74]
LM16	Processed arabinan–rhamnogalacturonan (RG)-I domain	[73]
LM6	1,5-alpha-L-arabinan	[75]
LM9	Feruloylated galactan	[76]
	*Hemicelluloses*	
LM21	β-linked mannan polysaccharides of plant cell walls	[77]
LM25	Xyloglucan	[78]

### Scanning electron microscopy

Small pieces of the calli with visible embryos were fixed in 3% (v/v) GA in 0.1M PBS (pH 7.2) for 2 h at RT. Next, the callus samples were rinsed 3×10 min in 0.1M PBS and postfixed in 1% aqueous OsO_4_ for 2 h at RT in the dark. After fixation, the samples were washed in PBS as described above and dehydrated in an ethanol series (30%, 50%, 70%, 80%, 90%, 95%, 100%) for 10 min each, followed by replacing the ethanol with acetone. The dehydrated samples were dried with a CPD 2 critical-point drier (Pelco) using liquid carbon dioxide, mounted on aluminium stubs with double-sided adhesive carbon tape and sputter-coated with a 20 nm film of gold in a SC-6 sputter coater (Pelco). After processing, the samples were observed using a Hitachi SU 8010 field emission scanning electron microscope (Hitachi High-Technologies Corporation) at 5 kV and 10 kV accelerating voltage with a secondary electron detector.

## Results

### General morphology and histology of the embryogenic calli

**Fig 1:**
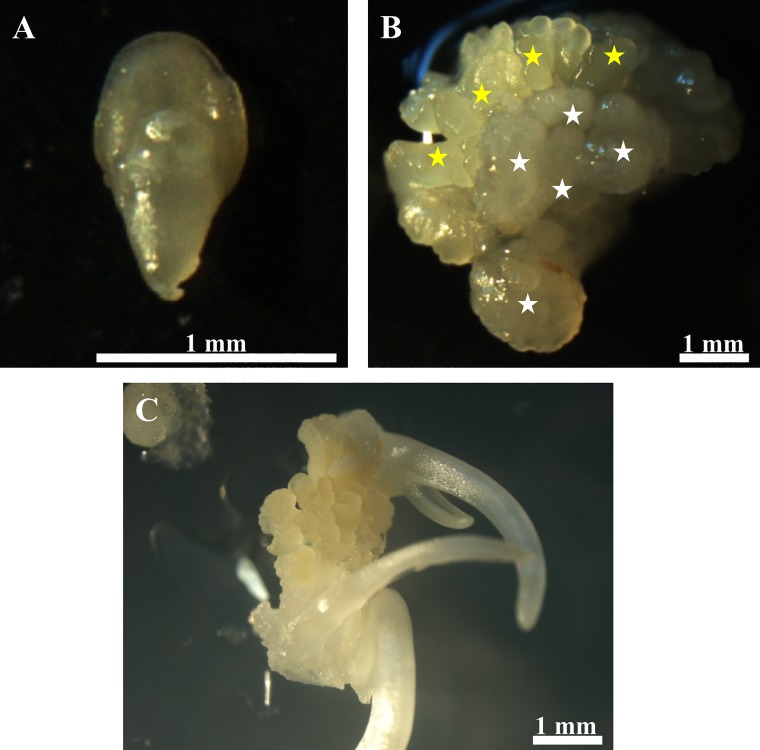
Morphology of selected structures in an *in vitro* Brachypodium culture. (A) Immature embryos. (B) Embryogenic callus–embryogenic masses marked by yellow asterisks; vitreous and friable callus indicated by white asterisks). (C) Spontaneous somatic embryogenesis seven day after cultivation on the CIM medium.

Embryogenic calli were obtained from immature zygotic embryos whose size varied from 0.6 to 0.8 mm wide (Fig 1A). The calli appeared on the medium with 2,4-D two weeks after cultivation. Noticeably, not all of the immature embryos had the capacity to produce an embryogenic callus; this was the case for about 70% of the embryos. The embryogenic calli were represented by two distinct categories, one of which was vitreous and friable (Fig 1B; white asterisks), while the other was a compact callus that was represented by embryogenic masses that were yellowish in colour (Fig 1B; yellow asterisks). The embryogenic masses appeared three weeks after cultivation during the first passage and then from the 7^th^ to the 10^th^ day of each passage (one passage took three weeks) after the callus was transferred to a new medium. The formation of somatic embryos was observed at the middle/end of each passage and developing coleoptiles could easily be observed during this process (Fig 1C).

**Fig 2:**
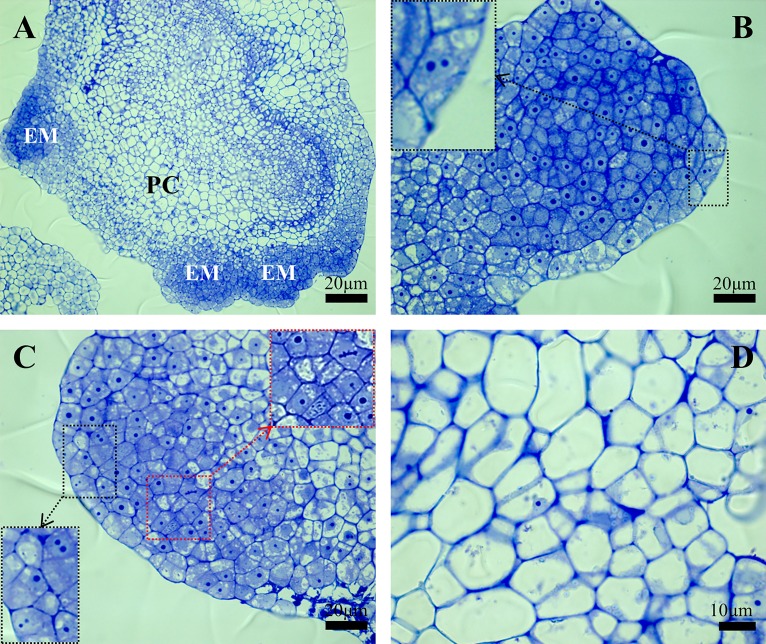
Histology of the Brachypodium embryogenic callus on the 7^th^ day after cultivation. (A) General view through the histological section with embryogenic masses (EM) and parenchymatous cells (PC). (B) and (C) Close-up of embryogenic masses, a black dotted square with an arrow shows examples of the cells that contain nuclei with two nucleoli; a red dotted square with an arrow indicates dividing cells. (D) Sector of callus that contains parenchymatous cells.

Histological analysis of the calli on the 7^th^ day after cultivation revealed the presence of different cell types (Fig 2A). Young, embryonic cells had a poorly developed vacuole system, dense cytoplasm and well-visible nuclei that usually had one and occasionally two nucleoli (Fig 2B and 2C; red arrows). Mitoses in these cells were also occasionally observed (Fig 2C; yellow arrows). In contrast, the parenchymatous cells were larger and highly vacuolated and it was difficult to spot the nuclei in many of them and, if they were visible at all, they were usually located close to the cell walls (Fig 2D).

### SEM analysis of the embryogenic callus surface

**Fig 3:**
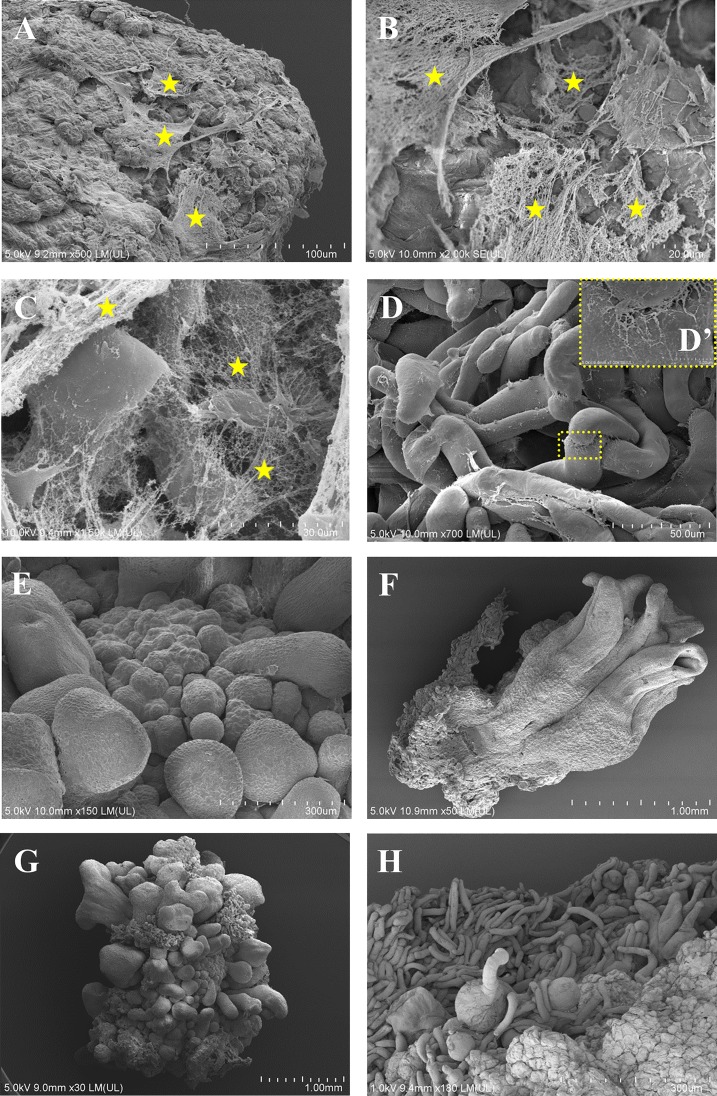
SEM images of the Brachypodium embryogenic callus surface pattern. A callus on the 7^th^ (A-E) and 21^st^ (F-H) days after cultivation. (A-C)–embryogenic masses covered by an extracellular matrix surface network (ECMSN is indicated by yellow asterisks): note the fibrillar and membranous structure. (D) Parenchymatous cells; high-magnification insert (D’) shows the contact between these cells. (E) Developing somatic embryos. (F) Developed multiple coleoptiles. (G) and (H) a callus on the 21^st^ day after cultivation showing the absence of ECMSN on the surface.

Observations under a scanning electron microscope allowed the changes that appeared during the callus cultivation to be understood in more detail. These were performed on the 7^th^ and the 21^st^ day after cultivation, which were the days when the most prominent changes were observed on the surfaces of the calli. At both time points, parenchymatous cells and newly formed embryoids were observed on the surfaces of the calli (Fig 3A–3E). On the 7^th^ day after cultivation, the embryogenic complexes were covered by a net-like ECMSN (Fig 3A–3C; yellow asterisks), which was in contrast to the parenchymatous cells (Fig 3D) and embryos in different stages of development (Fig 3E), which were smooth and did not have any fibrillar components on their surface. Interestingly, high-magnification analysis showed that fibrillary residues still occurred between the parenchymatous cells (Fig 3D’). The presence of developing embryos that had coleoptiles was observed on the 21^st^ day after cultivation (Fig 3F). On the 21^st^ day after cultivation the elements of ECMSN were absent (Fig 3G). It should be noted that large masses of parenchymatous cells could be observed at the end passage, i.e. on the 21^st^ day of callus cultivation (Fig 3H), comparing to the 7^th^ day after cultivation (Fig 3A).

### Immunodetection of AGP, extensin, pectin and hemicelluloses epitopes

The analysis of the specific cell wall epitope distribution was performed on the 7^th^ day after the passage to a fresh medium when embryogenic masses were clearly visible. We targeted 17 different antigens using the immunocytochemical approach of which five were against AGPs, three were against extensins, six detected pectic epitopes and two were against hemicelluloses. Only twelve of these antibodies gave positive results (Table 2).

**Table 2:** Summary of the immunocytochemical detection of selected antigens in embryogenic callus.

Antibody	Positive (+) or negative (-) reaction		Signal localisation	
		Cell wall compartments	Internal cell compartments	Callus surface
	*Arabinogalactan proteins*			
MAC207	+	+	+	+
JIM4	-	-	-	-
JIM8	-	-	-	-
JIM13	+	+	-	-
JIM16	+	+	+	-
LM2	+	+	+	+
	*Extensins*			
LM1	-	-	-	-
JIM11	+	-*	-	+
JIM12	+	-*	-	-
	*Pectins*			
LM19	*+*	-*	-	+
LM20	+	+*	-	+
LM13	-	-	-	-
LM16	+	-	+	-
LM6	+	+	+	-
LM9	-	-	-	-
	*Hemicelluloses*			
LM21	+	-	+	-
LM25	+	+	-	-

* signals were detected in the intercellular spaces for JIM11, JIM12, LM19 and LM20.

**Fig 4:**
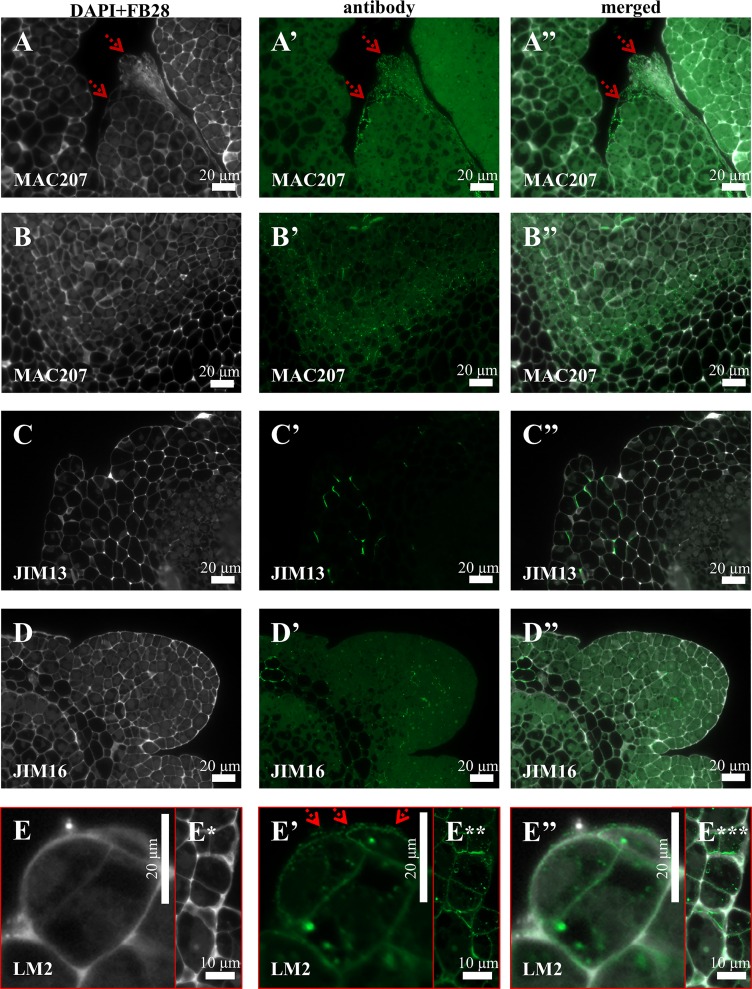
Immunolocalisation of arabinogalactans and arabinogalactan proteins in a Brachypodium embryogenic callus on the 7^th^ day after cultivation. (A)–(A”) and (B)–(B”) MAC207 in the cell walls of the dead cells on the callus surface (marked by red arrows) and parenchymatous cells, respectively. (C)–(C”) PC with JIM13. (D)–(D”) EM and PC with JIM16. (E)–(E”) EM and PC with LM2.

**Fig 5:**
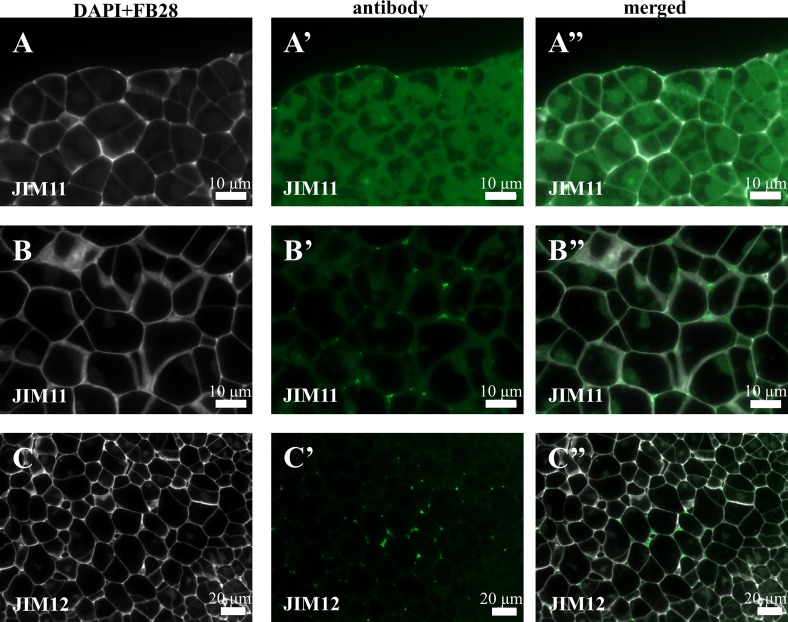
Immunolocalisation of extensins in a Brachypodium embryogenic callus on the 7^th^ day after cultivation. (A-A”) and (B-B”)–the surface of EM and PC with JIM11, respectively. (C-C”)–PC with JIM12.

Immunocytochemical detection of the antibodies directed to different AGPs revealed their diverse localisation in Brachypodium embryogenic callus. The MAC207 antibody was distributed intracellularly as well as in the cell walls (Fig 4A–4A” and 4B–4B”). Furthermore, this epitope marked the cell walls of the dead callus cells that were located on the callus surface (4A–4A”). The JIM13 epitope was localised in the cell walls of parenchymatous cells (PC) (Fig 4C–4C”), which differed from the cell wall and intracellular localisation of JIM16 and LM2 (Fig 4D–4D”, 4E–4E” and 4E*–4E***). Furthermore, the LM2 antibodies were localised on the surface of the embryogenic callus and seemed to be one of the ECMSN components (Fig 4E–4E”, ECMSN red arrows). Signals were not detected for antibodies such as JIM4 and JIM8. In the following analysis, three classes of antibodies against extensins–JIM11, JIM12 and LM1 (Fig 5A–5A”, 5B–5B” and 5C–5C”) were analysed. Weak signals of the JIM11 antibody were observed on the surface of embryogenic cells (5A–5A”). JIM11 and JIM12 were localised in intracellular compartments (Fig 5B–5B” and 5C–5C”). LM1 was not present in any of the types of callus cells.

**Fig 6:**
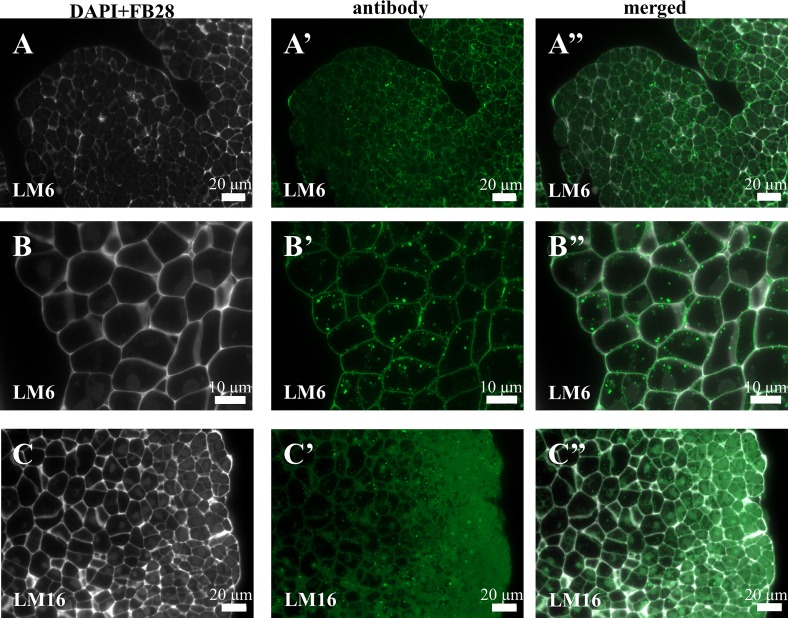
Immunolocalisation of pectins in a Brachypodium embryogenic callus on the 7^th^ day after cultivation. (A-A”) and (B-B”)–EM and PC with LM6, respectively. (C-C”)–EM and PC with LM16.

**Fig 7:**
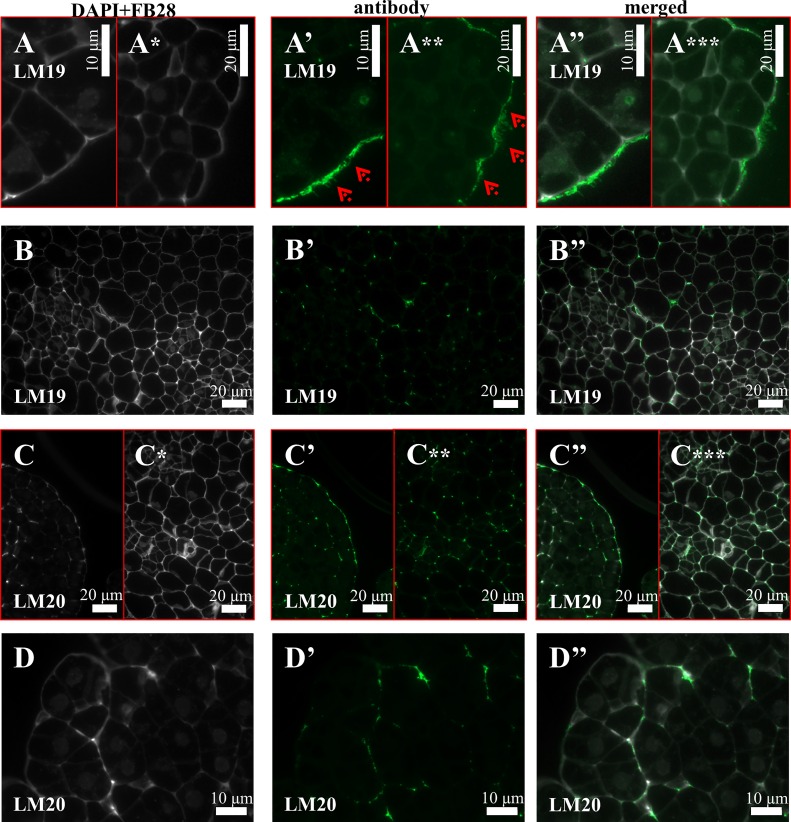
Immunolocalisation of pectins in a Brachypodium embryogenic callus on the 7^th^ day after cultivation. (A-A”) and (B-B”) EM and PC with LM19. (C-C”) and (D-D”)–EM with LM20.

Four of the six antibodies against pectic epitopes that were analysed gave positive signals. LM6 was localised in both cell wall and intracellular compartments (Fig 6A–6A” and 6B–6B”). The LM16 was exclusively localised intracellularly (Fig 6C–6C”). The distribution of the LM19 (Fig 7A–7A” and 7A*–7A***) and LM20 (7C-7C”) epitopes was very similar on the callus surface. The LM19 antibodies were part of the ECMSN (Fig 7A–7A” and 7A*–7A***, red arrows). Furthermore, the epitope that was recognised by the LM19 antibody was also detected in the cell wall of the parenchymatous cells (Fig 7B–7B”), while LM20 was also observed in the intercellular spaces (Fig 7C*–7C***) and in the cell walls between the newly formed embryogenic complexes (Fig 7D–7D”). No LM13 and LM9 antibodies were detected in any of the types of callus cells.

**Fig 8:**
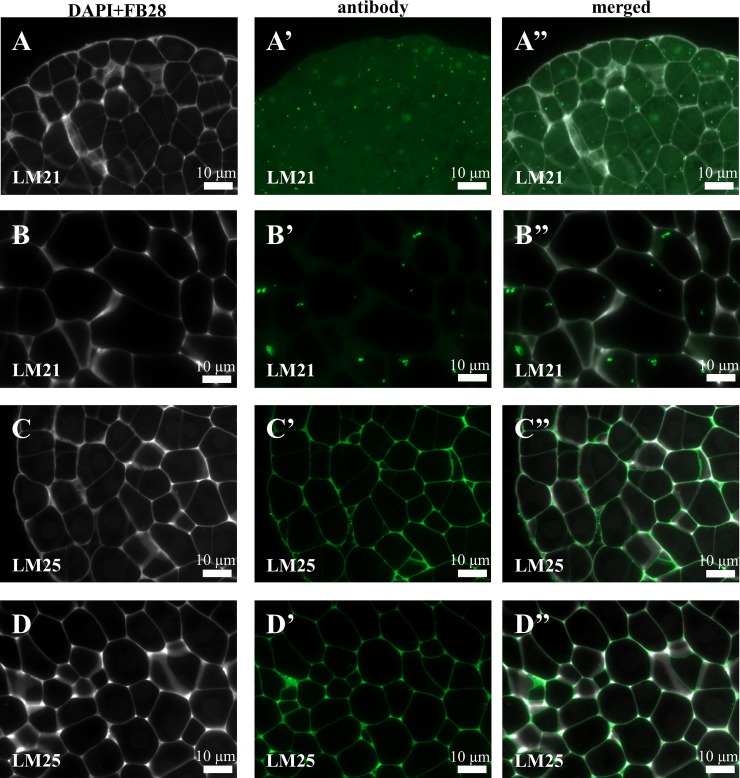
Immunolocalisation of hemicelluloses in a Brachypodium embryogenic callus on the 7^th^ day after cultivation. (A-A”) and (B-B”) EM and PC with LM21. (C-C”) and (D-D”)–EM and PC with LM25.

**Fig 9:**
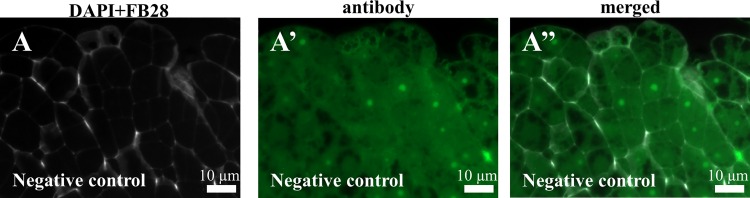
Negative control for the experiment with JIM13 antibodies. (A-A”)–no fluorescent signals were observed for these antibodies. Analogous results were obtained for all of the antibodies that were used.

Immunocytochemical detection of the two hemicelluloses that were analysed revealed their diverse localisation in the Brachypodium embryogenic callus. The LM21 antibodies only gave signals in the cell junctions of the embryogenic masses as well as in the parenchymatous cells (Fig 8A–8A” and 8B–8B”). The situation was different for the LM25 antibodies, which were visualised in the cell walls of both the embryogenic masses and parenchymatous cells (Fig 8C–8C”, 8D–8D”). The negative control for the JIM13 antibodies revealed the absence of any signals that were specific for these antibodies and was representative for all of the other tested antibodies (Fig 9A–9A”; analogous control experiments proved to be negative for the all of the antibodies that were used).

## Discussion

In dicotyledonous plants, embryogenesis of the callus can be induced not only from meristematic cells, but also from differentiated cells that have originated from leaf, stem or root tissues [21]. In contrast, in monocots only the meristematic cells have the potential to produce a callus. Although the callus in cereals can be obtained from the embryos, hypocotyls, root and stem apex, segments of young leaves and young inflorescences, effective plant regeneration is essentially limited to calli that are induced from young embryos and young inflorescences [19, 29].

As has already been demonstrated by several authors, the embryogenic calli of Brachypodium can be obtained from immature embryos [7, 8]. However, attempts to obtain calli from three-day-old coleoptiles, which is much faster than using immature embryos, were unsuccessful. We demonstrated that he calli obtained from young embryos and coleoptiles have an identical structure and morphogenic potential. According to Verdeil et al. [30], the embryogenic cells of *Cocos nucifera* are physically isolated from other cells by thick cell walls. They are also characterised by the presence of a nucleus with one nucleolus, and most of the chromatin is organised as euchromatin. In Brachypodium, we observed cells that were localised on the callus surface and that had features that are typical for embryogenetic cells, i.e. dense cytoplasm and mononucleolar nuclei that were centrally located and surrounded by small vacuoles.

The presence of ECMSN on the surface of embryogenic calli and its total absence on the surface of non-morphogenic calli has been demonstrated for many dicot species such as *Drosera rotundifolia* [29], *Papaver somniferum* [31], *Fagopyrum tataricum* [32] and *Actinidia deliciosa* [33]. Interestingly, ECMSN was observed on the surface of the non-morphogenic calli of *Helianthus tuberosus* that had been produced from different types of explants by Pilarska et al. [34]. These authors hypothesised that the formation of an extracellular net could be related to a stress response and protection against external factors that are specific to the culture conditions. For monocots, the presence of ECMSN has been demonstrated, for example on the morphogenic callus surface in *Zea mays* [29] and *Oryza sativa* [35], during androgenic plant regeneration from a *Triticum aestivum* anther callus [36] and in a suspension culture of *Panicum virgatum* [37]. ECMSN was present only on the surface of embryogenic cells in Brachypodium in all cases.

In the studies presented here, we also analysed the chemical composition of the cell wall, which is composed of various organic compounds including pectins, hemicelluloses, AGPs and extensins during the development of the embryonic mass. As was demonstrated by Samaj et al. [19], the chemical composition of ECMSN can differ significantly between monocotyledonous and dicotyledonous plants. However, studies on the protein ECMSN compounds during callo- and embryogenesis in monocots are still quite scarce.

Hydroxyproline-rich proteins (HRGPs) consist of four major classes of proteins: highly glycosylated AGPs, moderately glycosylated extensins, proline-rich proteins (PRPs) and lectins [38, 39]. The exact classification of HRGPs is still under debate. AGPs can be localised in the cell wall or on the surface of the cell wall, deposited into the intracellular compartments or secreted into the medium [40, 41]. Furthermore, AGPs are one of the ECMSN components that are pivotal for the correct cell division and expansion, programmed cell death, somatic embryogenesis and many other important aspects of plant cell function [41, 42]. Interestingly, among the analysed AGPs, JIM4, JIM8, JIM13 and JIM16 antibodies did not localise on the surface of the embryogenic culture of Brachypodium; they were either distributed intracellularly or in the cells walls or were totally absent. The only exceptions were for MAC207 and LM2-labelled AGP. MAC207 was localised on the callus surface but only in the cell walls of the dead cells and in the internal cell compartments of living cells. As has been revealed in many other studies, for example in *Daucus carota* [43, 44], *Trifolium nigrescens* [45] and *Centrarium erythraea* [46], the distribution of MAC-207 is highly diverse and depends on the type of cell, tissue and organ studied. The LM2-labelled epitopes were components of the ECMSN but were also localised in the cell wall and internal cell compartments.

Extensins are known to have different functions during both abiotic and biotic stress and play important roles in the response to wounding and pathogen infections [47–49]. However, there are only a few works demonstrating the localisations of extensins in a plant tissue culture. In their study on *Dactylis glomerata*, Zagorchev and Odjakova [49] demonstrated the localisation of JIM12-labelled proteins in a medium of a salt adapted embryogenic suspension culture and hypothesised that extensins could play roles in a stress response and in the determination of cell fate during somatic embryogenesis. When they observed a very high level of the surface expression of JIM11 and JIM20-labelled epitopes and very weak signals on the surface of non-embryogenic cells in the protocorm-like bodies of the *Phalaenopsis* tissue culture, Lee et al. [50] suggested that JIM11 and JIM20 HRGPs could be used as effective markers to survey the embryogenic competence in different orchid callus cultures. The LM11 and LM20 antibodies were also found in the cell walls and outer surface layer of embryogenic cells, proembryos and globular embryos in *Musa* spp. AAA, which was attributed to the fact that HRGPs play important roles in the process of the regeneration and germination of embryos during plant regeneration via somatic embryogenesis [51]. Interestingly, in the case of a Brachypodium culture, we revealed the presence of an extensin that is recognised by the JIM11 antibodies and the total absence of an extensin that is recognised by the JIM12 antibody on the surface of embryogenic cells. Nonetheless, both proteins were localised in the intracellular compartment of parenchymatous cells. It can be hypothesised that extensins could play important roles during the embryogenic mass formation and in stress response in a Brachypodium tissue culture. However, for a more precise understanding the function of extensins during the formation of the embryogenic mass, studies using inhibitors of HRGPs biosynthesis such as 3,4-dehydro-L-proline (3,4-DHP) would be required.

Pectins are an indispensable compound of the cell walls of both land and aquatic higher plants. They perform a variety of important biological functions, support the water regime and influence seed germination and plant growth and development. Pectins are also known to play protective roles in the interactions between plants and phytopathogens [52, 53]. To date, studies on the functions of pectins in monocots are very limited [34, 40, 54, 55]. In their study on androgenic cultures of wheat cultivars, Pilarska et al. [56] demonstrated that the xylogalacturonan-targeting LM8 antibody gave signals only in a peripheral cell and that the JIM5 antibody specifically detected methyl ester-rich pectins, in the cell walls of the callus parenchyma, thus suggesting the importance of pectic substances in wheat androgenesis, and possibly in the regulation of cellular adhesion. The high level of LM19 HG on the surface of the embryogenic masses that was observed in our study provides another example of the presence of pectins in the extracellular matrix and are consistent with previous studies describing the chemical composition of the ECMSN [45, 57, 58]

It is worth mentioning that the LM6 antibody generally occurs as a side chain of RG-I but in the case of the moss *Physcomitrella patens* it was attributed to the arabinogalactan proteins [59] and in the ‘C-Fern’ gametophytes and sporophytes of *Ceratopteris richardii*, observations suggest that the LM6 epitope may be associated with AGPs rather than [60]. Thus, our findings concerning the presence of this epitope require further studies to answer the question of whether it is attributed to pectins or arabinogalactan proteins. The process of somatic embryogenesis is associated with a number of changes within the cell wall macromolecules including hemicelluloses [21]. Hemicelluloses are the second most abundant polysaccharides in plant cell walls after cellulose. These polymers, together with pectins (also lignins) form the complex cell wall matrix between the cellulose microfibril network [61]. Depending on the main type of sugar residues, hemicelluloses are divided into mannans, xylans, xyloglucans and mixed linkage β-glucans. Hemicelluloses are present in greater amounts in the secondary cell wall than in the primary walls of both monocot and dicot species. Moreover, monocots have significantly more hemicellulose and less pectins than dicots. Galactomannans and galactoglucomannans are structurally important components of the cell walls and are also an important source of storage polysaccharides [62, 63]. In our work, we found the signals of the LM21 antibodies bound to mannans only in the internal cell compartments, which may be linked with their storage function in the embryogenic callus of Brachypodium. Xyloglucans are the most common hemicelluloses in dicot primary cell walls but they are scarce in monocots [64]. The primary function of xyloglucan is the formation of a network with cellulose microfibrils in the cell walls but some reports have also demonstrated its role in cell signalling [65] and as a storage material in seeds [63]. Our work demonstrated that the hemicellulose that is recognised by the LM25 antibodies (for xyloglucan) was present in the cell walls and the antibody that was used for its detection gave the most abundant signals among all of the antibodies that were used.

For the first time, we have demonstrated in this study that AGPs and pectins are the components of the ECMSN in the embryogenic callus of Brachypodium. We found that the main characteristic of cell wall components of Brachypodium embryogenic calli are AGPs epitopes that are recognised by the JIM16 and LM2 antibodies, an extensin epitope that is recognised by the JIM11 antibody and a pectic epitope that is recognised by the LM6 antibody. Of potentially practical importance is that *in vivo* and *in vitro* analyses of the cell wall chemical composition in Brachypodium demonstrated the presence of compounds that are similar to those that are present in the grass species that are important for pasture or bioenergy production [66]. Our findings demonstrate that a Brachypodium tissue culture can provide a good model system to reveal the functions of HRGPs and pectins during the formation of the embryogenic mass and somatic embryogenesis in grasses. However, for a more precise understanding of their functions during the formation of the embryogenic mass, studies using inhibitors of HRGPs and pectin biosynthesis will be required. Taking into account the present impact of Brachypodium as a well-established model organism, such studies are worth considering.
